# Anticipating Urinary Retention Following Total Hip and Total Knee Replacements

**DOI:** 10.5704/MOJ.2011.017

**Published:** 2020-11

**Authors:** Y Hamed, A Ramesh, R Taylor, R Michaud

**Affiliations:** 1 Department of Trauma and Orthopaedics, Furness General Hospital, Barrow-in-Furness, United Kingdom; 2 Department of Medicine, Royal Bolton Hospital, Bolton, United Kingdom

**Keywords:** lower limb, arthroplasty, urinary retention, catheterisation

## Abstract

**Introduction:**

Urinary retention is a widely recognised postoperative complication. Although anecdotally lower limb arthroplasty is linked with high rates of urinary retention, there are no current accepted standards for determining which patients are at higher risk and should therefore be offered intra operative catheterisation.

**Materials and Methods:**

One hundred patients, 55 females and 45 males, who underwent uncomplicated total hip or total knee replacements at Furness General Hospital were recruited between January and April 2017.

**Results:**

Post-operative urinary retention was seen frequently, with 38 patients (38%) requiring post-operative catheterisation. Twenty-one males (46%) developed postoperative retention compared to 17 (30%) of females, representing a statistically significant increase in risk seen in male patients. (p 0.009). Post-operative urinary retention requiring catheterisation was associated with increasing age, with those over 75 years having a significantly higher risk than those less than 75 years irrespective of gender (p 0.04). There was no significant difference in urinary retention rates between patients who had general (n=21) or spinal anaesthetic (n=79) with 33% of GA patients and 39% of spinal anaesthetic patients requiring catheterisation (p 0.17).

**Conclusion:**

There are increased rates of urinary retention seen in lower limb arthroplasty patients than those described in the general surgical population, with male patients and all those over 75 years of age having a significantly higher risk. Clinically, it may therefore be sensible to consider offering routine intra operative catheterisation to this cohort of patients.

## Introduction

Post-operative urinary retention (POUR) is a recognised surgical complication which can have serious consequences if not swiftly and appropriately managed^1^. In addition to being a painful and unpleasant experience, patients who develop POUR are at risk of acute kidney injury, delirium, cardiac arrhythmias and long term detrusor dysfunction which can pose a significant risk to both short and long term health^1^. Studies have reported a significantly increased risk of POUR in patients undergoing lower limb orthopaedic procedures when compared to the general surgical population, especially those undergoing hip or knee arthroplasty, with POUR incidence rates reported at around 5% in the general surgical population compared to 77.8% seen following hip arthroplasty procedures^2,3^. As the number of lower limb joint replacement surgeries are steadily increasing, with the UK national joint registry recording an increase of over 20,000 replacements per annum, POUR is consequentially developing as significant complication effecting orthopaedic practice.

POUR is treated through insertion of a urinary catheter to allow bladder drainage until detrusor function recovers, the development of POUR can therefore be avoided through intra operative catheterisation of patients^3^. As catheterisation itself carries associated risks, including infection, bladder injury, urethral trauma and stricture development^4^ routine intra operative catheterisation should be reserved for patients whose risk of developing POUR outweighs the risks associated with urinary catheterisation. Despite the increased prevalence of POUR in lower limb orthopaedic procedures and the availability of an effective strategy to avoid this complication, there is currently no clear guidance available to effectively determine which patients should be offered routine intra-operative catheterisation whilst undergoing lower limb arthroplasty operations.

This study aimed firstly to determine the true incidence of post-operative urinary retention following lower limb arthroplasty. Secondly, to determine which factors increase the risk of post-operative urinary retention in these patients to identify those who would benefit from routine intra operative urinary catheterisation, providing guidance for current clinical management.

## Materials and Methods

One hundred patients, 55 females and 45 males, who underwent uncomplicated total hip or total knee replacements at Furness General Hospital were recruited between January and April 2017.

Patients excluded from the study were those who required post-operative urinary catheterisation for medical reasons, for example those who required close fluid balance monitoring due to co-existing co morbidities. Patients undergoing revision arthroplasty surgery or unicompartment knee replacement were also excluded due to the added technical complexity of and narrow subset of patients suitable for these procedures.

Data was collected from both electronic and paper preoperative assessment reports, intra-operative anaesthetic charts, operation notes, drug charts, post-operative electronic and paper clinical notes and discharge summary reports. In patients who developed post-operative urinary retention electronic records were accessed to confirm that catheterisation was as required and the difficulty of the catheterisation procedure itself. Risk variables assessed included gender, age, history of prostatism, smoking status, social status, type of anaesthesia, American Society of Anaesthesiologist (ASA) grade and length of hospital stay.

Data was recorded and complied into a database using Microsoft excel and statistical analysis was performed using SPSS software.

## Results

Of the 100 patients recruited 38 (38%) developed POUR requiring the insertion of a urinary catheter. Those who developed POUR had an average increase in hospital stay length of one day compared to their non-retention counterparts.

**Fig. 1:**
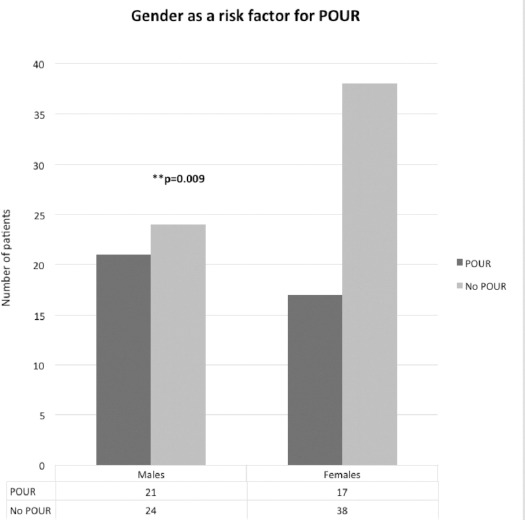
The incidence of POUR following lower limb arthroplasty

There was a statistically significant increase in POUR seen in male patients with 21 males (46%) developing postoperative retention compared to 17 females (30%) (p 0.009 unpaired T test) (Fig. 1).

Among the 45 male patients, only one had a confirmed diagnosis of benign prostatic hypertrophy and did subsequently develop POUR, post-operative catheter insertion in this patient was document as being simple and un-complicated.

**Fig. 2:**
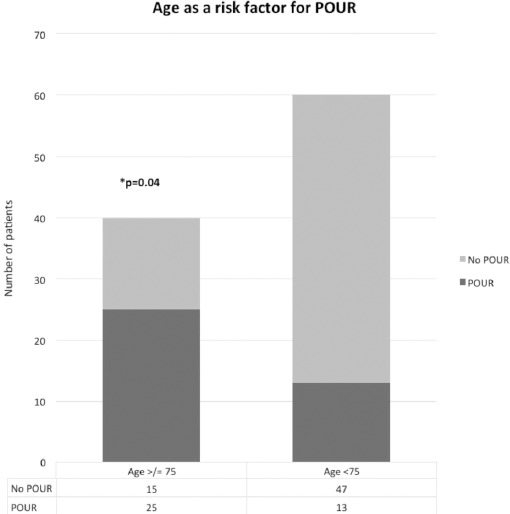
POUR following lower limb arthroplasty is significantly is significantly higher in male patients. associated with increasing age.

**Fig. 3:**
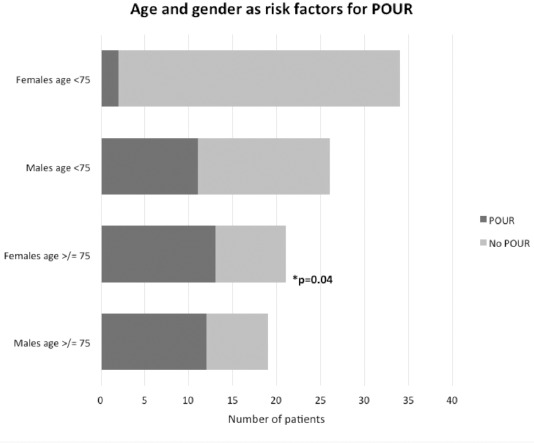
POUR following lower limb arthroplasty is significantly associated with increasing age, irrespective of gender.

Post-operative urinary retention was significantly associated with increasing age, with those over 75 years (n=40) having a significantly higher risk of developing POUR than those less than 75 years (n=60), irrespective of gender (p 0.04 Chi Squared test) (Fig. 2, Fig. 3).

There was no significant difference in urinary retention rates between patients who had general anaesthesia (n=21) or spinal anaesthesia (n=79) with 33% of GA patients and 39% of spinal anaesthetic patients requiring catheterisation (p 0.17 unpaired T Test).

The majority of patients, 67% (n=67) were ASA grade two, of the remaining 23% (n=23) were ASA grade three and 10% (n=10) were ASA grade one. There was no statistically significant variation in risk of POUR seen with increasing ASA grade.

## Discussion

Results from this study strengthen the association of POUR with lower limb arthroplasty. Previous research^1,3,5^ had highlighted the fact that surgery in general, as well lower limb arthroplasty may put patients at a higher risk of developing POUR. Results from our study definitively demonstrate that POUR is significantly increased in lower limb arthroplasty procedures, with risk of development five times higher than those seen in the general surgical population.

Although previous studies have aimed to develop scoring systems to predict those at higher risk of POUR they have only included data from male patients^6^. One study concluding that all male patients over 70 years of age should be considered for intra-operative catheterisation when undergoing lower limb joint replacement surgery^7^. This lack of inclusion of female data in previous studies can be attributed to the fact that male patients are assumed to have a naturally higher risk of POUR development due to the presence of, known or unknown, prostatism. By including both genders, with almost equal distribution, in this study we have been able to definitively demonstrate that male gender is in fact a significant risk factor associated with the development of POUR but, crucially, that patients above 75 years of age, regardless of gender, are also at a significantly increased risk of developing POUR.

Although the current age at which patients are clinically classed as elderly in the UK is 65 years, those above the age of 65 did not have a statistically significant increased risk of POUR as compared to those below this age bracket (p 0.08 Chi Squared test). Due to this negative finding further analysis of data in patient age groups, increasing at 5 years increments, demonstrated crucially that patients in the age group of 75 and above (n=40), have a significantly higher risk of developing POUR than patients below 75 years (n=60). This significant increase in risk was also shown to be present irrespective of gender (p 0.04 Chi Squared test) (Fig. 2, Fig. 3). This is clearly potentially of clinical significance as those over the age of 75 may therefore benefit from routine catheterisation.

This study has shown that when considering the development of a risk stratification model to determine which patients would benefit from routine catheterisation it is vital not to overlook the assessment of female patients as they also remain at significant risk of developing POUR with increasing age. Other parameters, such as ASA grade and the type of anaesthesia used, had no significant effect on the risk of POUR development and should therefore not be considered when assessing risk of POUR.

It is clear from the results of this study that POUR is a significant issue effecting patients following lower limb arthroplasty. Ultimately as routine intra-operative catherisation of patients provides a simple avenue to avoid to POUR, and a clear cohort of patients at increased risk has been identified by this study, a change in practice through offering all male patients and those 75 years and older routine intra-operative catheterisation may be of significant clinical benefit. However, one should bear in mind that urinary catherisation can be associated with potential risks^8,9,10^ hence a clear discussion should take place with patients in the pre-operative consenting process.

## Conclusion

Patients who undergo lower limb arthroplasty surgery are at a high risk of developing post-operative retention of urine when compared to the general surgical population. Patients who are male and those who are 75 years of age and above have a significantly higher risk of developing POUR. Currently, there is no consensus on who should be offered intra-operative catheterisation but clinically it may be sensible to consider offering routine intra operative catheterisation to this cohort of patients.
